# Comparative Evaluation of Clinical and Immunonutritional Risk Scores for Predicting Contrast-Associated Acute Kidney Injury in Emergency Patients

**DOI:** 10.3390/diagnostics15222842

**Published:** 2025-11-10

**Authors:** Meliha Fındık, Muhammet Çakas, Uğur Demir

**Affiliations:** 1 Department of Emergency Medicine, Balikesir University, 10145 Balikesir, Türkiye; u36104@hotmail.com; 2 Bingöl Genç State Hospital, 12500 Bingöl, Türkiye; muhammetcakas@gmail.com

**Keywords:** contrast-associated acute kidney injury, pre-CT AKI score, risk prediction, nephropathy, biomarkers, emergency medicine

## Abstract

**Background:** Contrast-associated acute kidney injury (CA-AKI) is a clinically important complication following contrast-enhanced computed tomography (CT), particularly in emergency department (ED) populations. While several risk scores have been proposed, their comparative performance in ED-based imaging remains uncertain. **Methods:** This retrospective single-center study included 472 adult patients who underwent contrast-enhanced CT between November 2023 and November 2024. Patients with end-stage kidney disease, renal transplantation, baseline eGFR < 30 mL/min/1.73 m^2^, or incomplete laboratory data were excluded. CA-AKI was defined as an increase in serum creatinine ≥ 0.3 mg/dL or ≥25% within 48–72 h after contrast exposure in the absence of alternative causes. The Mehran score, Pre-CT AKI score, and immunonutritional indices—including the Prognostic Nutritional Index (PNI), Osaka Prognostic Score (OPS), and Glasgow Prognostic Score (GPS)—were calculated. Predictive performance was evaluated using logistic regression and receiver operating characteristic (ROC) curve analyses. **Results:** The incidence of CA-AKI was 2.1% (n = 10). Patients who developed CA-AKI were older and had more comorbidities, particularly chronic kidney disease, diabetes, and cardiovascular disease. In univariate analysis, baseline eGFR, Pre-CT AKI score, and PNI were significantly associated with CA-AKI. Multivariate logistic regression identified baseline eGFR and PNI as independent predictors. The Pre-CT AKI score demonstrated the highest discriminative ability (AUC = 0.87), outperforming the Mehran score (AUC = 0.74). PNI provided complementary prognostic value (AUC = 0.71), whereas OPS and GPS did not reach statistical significance. **Conclusions:** In ED patients undergoing contrast-enhanced CT, the Pre-CT AKI score was the most accurate predictor of CA-AKI, while PNI offered additional prognostic information reflecting immunonutritional vulnerability. The Mehran score showed moderate usefulness, whereas OPS and GPS were less applicable. Incorporating multifactorial models that integrate clinical, hemodynamic, and immunonutritional factors may improve early risk stratification and guide preventive strategies for CA-AKI in emergency settings.

## 1. Introduction

Contrast-associated acute kidney injury (CA-AKI), previously referred to as contrast-induced nephropathy (CIN), is one of the most frequent causes of hospital-acquired acute kidney injury and is associated with prolonged hospitalization, increased healthcare costs, and elevated morbidity and mortality—particularly in emergency department (ED) populations undergoing contrast-enhanced imaging [1]. Recent studies, such as Cho et al. (2022), have emphasized the distinction between CIN and CA-AKI and advocated for risk-based, context-aware prevention strategies within ED workflows [2]. Early identification of high-risk patients is therefore essential to enable preventive measures such as adequate hydration, minimizing contrast dose, and avoiding nephrotoxic agents. Recent consensus guidelines have underscored the need for standardized terminology and multifactorial, risk-based prevention approaches [3].

In clinical practice, the baseline estimated glomerular filtration rate (eGFR) remains the most widely used parameter to guide prophylactic measures. However, ED environments are inherently time-constrained and clinically heterogeneous, limiting the accuracy of single-parameter screening. Wu et al. (2022) demonstrated that the risk of CA-AKI after contrast-enhanced CT increases progressively with chronic kidney disease (CKD) stage, supporting a graded, stage-aware stratification rather than fixed eGFR thresholds [4]. The American College of Radiology (ACR)–National Kidney Foundation consensus and the 2024 KDIGO guideline both recommend structured risk assessment rather than dichotomous cutoffs, emphasizing that while the absolute risk of CA-AKI is generally low, it rises substantially in patients with advanced CKD or hemodynamic instability [3]. Importantly, differentiating true contrast-related nephrotoxicity from illness-associated AKI remains a persistent methodological challenge in retrospective studies, suggesting that some reported risk models may reflect overall illness severity rather than contrast-specific effects [1].

Historically, the Mehran score, developed for coronary angiography patients, has served as the prototypical multivariable risk tool for predicting contrast-related nephropathy [5]. Although it has shown predictive value beyond cardiac contexts [6], its accuracy in ED-based contrast-enhanced CT remains limited. Recently, the Pre-CT AKI score was introduced and externally validated as a CT-specific risk model, showing moderate discrimination (AUC ≈ 0.71) and practicality for use in acute care settings [1]. Nevertheless, its generalizability across diverse ED populations and imaging modalities remains uncertain. Beyond score-based triage, emerging biomarkers such as neutrophil gelatinase-associated lipocalin (NGAL) have been shown to detect subclinical kidney injury early and may complement existing risk scores in ED-relevant settings [7].

Beyond traditional clinical scores, immunonutritional indices such as the Prognostic Nutritional Index (PNI), the Osaka Prognostic Score (OPS, based on CRP, albumin, and lymphocyte count), and the Glasgow Prognostic Score (GPS/mGPS) have demonstrated prognostic value in multiple clinical contexts and were recently linked to CIN/CA-AKI risk in coronary cohorts [8,9]. Similarly, Yıldız et al. (2025) reported that inflammation-based composite indices such as the systemic immune-inflammation index (SII) and systemic inflammation response index (SIRI) are independently associated with CI-AKI, highlighting the contribution of systemic inflammation to renal vulnerability [10]. These indices are derived from routine laboratory tests, which are inexpensive and widely available, making them appealing for ED practice. However, their incremental predictive value beyond baseline renal function and CT-specific scores in emergency imaging remains uncertain.

This study aims to compare multivariable clinical tools (Mehran and Pre-CT AKI scores) and immunonutritional indices (PNI, OPS, and GPS) with single-parameter screening methods in identifying patients at risk of contrast-associated acute kidney injury (CA-AKI) in the emergency department. By integrating clinical, hemodynamic, and immunonutritional markers, this study aims to establish a more comprehensive, personalized, and practical framework for assessing the risk of contrast-induced kidney injury in emergency medicine.

## 2. Materials and Methods

### 2.1. Study Design and Setting

This retrospective cohort study was conducted at the Department of Emergency Medicine, Balıkesir University, between 1 November 2023, to 1 November 2024. The study protocol was approved by the local ethics committee (Approval No: 2025/12). Informed consent was waived due to the study’s retrospective design.

### 2.2. Study Population

**Figure 1:**
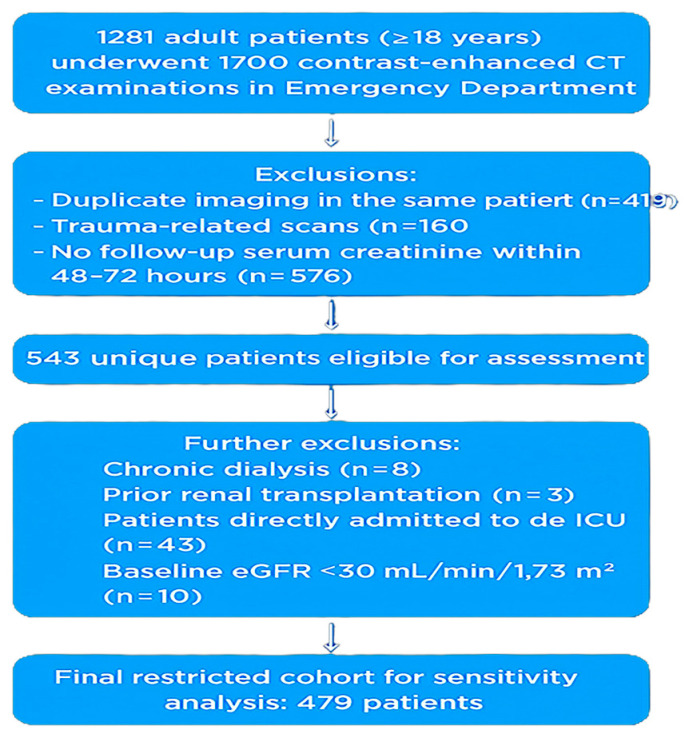
Flow Diagram of Patient Selection.

We initially screened 1281 consecutive adults (≥18 years) who underwent 1700 contrast-enhanced CT examinations during their ED visit. We then excluded duplicate imaging in the same patient (n = 419), trauma-related scans (n = 160), and cases without a follow-up serum creatinine within 48–72 h (n = 576), leaving 543 unique patients. Patients on chronic dialysis, with prior renal transplantation, or with baseline eGFR < 30 mL/min/1.73 m^2^ were excluded. The final main cohort comprised 522 patients. For sensitivity analyses, we additionally exclude patients admitted directly to the ICU to mitigate confounding from multifactorial AKI; the restricted cohort size is reported in the Results. A priori, a power analysis with G*Power 3.1 (assumed CIN incidence 7%, α = 0.05, power = 0.80) indicated a minimum required sample size of 27; our final sample far exceeded this threshold. The patient selection process is summarized in Figure 1.

### 2.3. Contrast Administration Protocol

All CT examinations were performed using the same nonionic, low-osmolar contrast medium (Iohexol, KOPAQ^®^ 350 mgI/mL; Koçak Farma, Istanbul, Turkey). Contrast volume was determined according to the imaging protocol: thoracic CT (70 mL), abdominal CT (70 mL, angiographic phase included), peripheral CT angiography (40 mL), brain and carotid CT angiography (80 mL), thoracoabdominal CT angiography (90 mL), and pulmonary CT angiography (90 mL). All injections were administered via peripheral venous access using a power injector, followed by a 30–40 mL saline flush.

### 2.4. Definitions

CIN was defined as an increase in serum creatinine ≥ 0.3 mg/dL or ≥25% from baseline within 48–72 h after contrast exposure, in the absence of alternative causes. Advanced CKD was defined as eGFR < 30 mL/min/1.73 m^2^ (excluded from the main cohort). ICU patients were analyzed separately in sensitivity models, given their high risk of multifactorial AKI.

### 2.5. Data Collection

Demographic, clinical, and laboratory data were extracted from the hospital’s electronic medical record system. Variables included age, sex, vital signs, comorbidities, and baseline laboratory results (hemoglobin, WBC count, neutrophil and lymphocyte counts, albumin, CRP, creatinine, and eGFR). CRP and albumin are routinely measured in our ED for patients with infections, systemic illnesses, or suspected inflammatory conditions, which allowed these variables to be included in the analysis. Laboratory values were recorded at baseline and at 48–72 h after exposure to the contrast agent. All records were reviewed and manually verified by the investigators. Patients with missing outcome data (i.e., no follow-up creatinine within 72 h) were excluded from the analysis.

The definitions of the Mehran Risk Score, Pre-CT AKI score, Osaka Prognostic Score (OPS), Glasgow Prognostic Score (GPS/mGPS), and Prognostic Nutritional Index (PNI) were based on previous studies [1,5,11,12,13]. Detailed descriptions of the included parameters, scoring methods, and clinical interpretations for each index are presented in Table 1.

The OPS was calculated using three parameters: C-reactive protein (CRP; ≤10.0 mg/L = 0 point, >10.0 mg/L = 1 point), albumin (ALB; ≥3.5 g/dL = 0 point, <3.5 g/dL = 1 point), and total lymphocyte count (TLC; ≥1600/μL = 0 point, <1600/μL = 1 point). The total OPS score was calculated by summing these components (range: 0–3), reflecting the patient’s inflammatory and nutritional status.

The GPS was determined according to CRP and albumin levels: patients with CRP ≤ 10 mg/L and albumin ≥ 3.5 g/dL were assigned a score of 0; those with either CRP > 10 mg/L or albumin < 3.5 g/dL received a score of 1; and those with both CRP > 10 mg/L and albumin < 3.5 g/dL were scored as 2. The PNI was computed as 10 × albumin (g/dL) + 0.005 × lymphocyte count (/mm^3^), representing a composite index of nutritional and immunological status.

The Pre-CT AKI and Mehran scores were calculated according to their original definitions to evaluate the risk of contrast-induced nephropathy (CIN). These indices were used to compare the predictive performance of clinical, nutritional, and inflammatory parameters in identifying patients at increased risk of CIN.

The primary outcome was the incidence of CIN after contrast-enhanced CT. Secondary outcomes included identification of risk factors, evaluation of the predictive performance of clinical and immunonutritional scores, and assessment of their incremental value over baseline eGFR. Sensitivity analyses were conducted, excluding patients with advanced CKD and those admitted to the ICU.

### 2.6. Statistical Analysis

All analyses were performed using SPSS version 25.0 (IBM Corp., Armonk, NY, USA). Continuous variables were expressed as mean ± standard deviation (SD) or median with interquartile range (IQR), as appropriate, and categorical variables as frequencies and percentages. Normality was assessed using the Shapiro–Wilk test. Group comparisons were performed using the Student’s *t*-test or Mann–Whitney U test for continuous variables, and chi-square or Fisher’s exact test for categorical variables.

Multivariable logistic regression was used to identify independent predictors of CIN, with results reported as odds ratios (OR) and 95% confidence intervals (CI). Model calibration was assessed with the Hosmer–Lemeshow goodness-of-fit test. Discriminative ability of each risk score (Mehran, Pre-CT AKI, PNI, OPS, GPS) was evaluated using receiver operating characteristic (ROC) curve analysis. The Youden index was used to identify optimal cut-off values, as it balances sensitivity and specificity, which is particularly relevant for risk prediction in the ED setting. A two-tailed *p*-value < 0.05 was considered statistically significant.

All materials, data, and statistical protocols related to this study are available from the corresponding author upon reasonable request. No custom computer code was developed; all analyses were performed using SPSS version 25.0.

## 3. Results

A total of 479 patients were included in the final analysis, of whom 10 (2.1%) developed contrast-induced nephropathy (CIN). Patients with CIN were significantly older than those without CIN (79.7 ± 7.4 vs. 57.5 ± 19.1 years, *p* < 0.001). The sex distribution did not differ significantly between groups (male: 80.0% vs. 49.5%, female: 20.0% vs. 50.5%; *p* = 0.112). In terms of vital signs, the CIN (+) group had a higher heart rate (98.8 ± 19.6 vs. 89.4 ± 19.4 bpm, *p* = 0.119) and lower oxygen saturation (94.6 ± 3.3% vs. 96.0 ± 3.9%, *p* = 0.062), although these differences did not reach statistical significance.

Regarding comorbidities, the prevalence of hypertension (50.0% vs. 34.8%, *p* = 0.506), diabetes mellitus (40.0% vs. 24.3%, *p* = 0.442), stroke (20.0% vs. 4.1%, *p* = 0.098), and malignancy (10.0% vs. 6.8%, *p* = 1.000) was numerically higher in patients with CIN, but none reached statistical significance, likely due to the small number of CIN cases. Baseline demographic and clinical characteristics are summarized in Table 2.

When stratified by hospital outcomes, CIN incidence was higher among patients admitted to the ward compared with those discharged (3.0% vs. 0.7%, *p* = 0.019) (Table 3).

When stratified by CT modality, the incidence was 6.6% following pulmonary CT angiography and 1.6% after abdominal CT, whereas no CIN cases were observed in other CT types (Table 4). The difference between imaging modalities was statistically significant (*p* < 0.001).

When stratified by primary diagnoses, CIN incidence was highest in patients with decompensated heart failure (12.5%) and choledocholithiasis (7.7%), while pneumonia, cholecystitis, ileus, urinary tract infection, myocardial infarction, and pulmonary embolism were associated with lower but notable incidences. No CIN cases were observed among patients with other diagnoses (Table 5).

The comparison of laboratory findings between patients who developed CIN and those who did not is summarized in Table 6. The overall mean serum albumin level of the cohort was 38.38 ± 4.78 g/L. Patients who developed CIN had significantly lower albumin levels compared to those without CIN (35.10 ± 3.18 vs. 38.45 ± 4.79 g/L, *p* = 0.028). Similarly, lymphocyte counts and prognostic nutritional index (PNI) values were significantly reduced in the CIN (+) group (*p* = 0.015 and *p* = 0.028, respectively).

Baseline renal parameters also differed between the groups. Patients with CIN had higher baseline serum creatinine (1.12 ± 0.24 vs. 0.92 ± 0.28 mg/dL, *p* = 0.026) and lower baseline eGFR (52.20 ± 16.30 vs. 84.52 ± 25.04 mL/min/1.73 m^2^, *p* < 0.001). At follow-up, these differences became more pronounced, with creatinine rising markedly in CIN (+) patients (2.02 ± 1.07 vs. 0.83 ± 0.24 mg/dL, *p* < 0.001) and eGFR declining substantially (29.90 ± 13.11 vs. 90.98 ± 23.39 mL/min/1.73 m^2^, *p* < 0.001).

No statistically significant differences were observed in CRP, hemoglobin, total white blood cell count, or neutrophil counts between the two groups.

In univariate logistic regression, baseline eGFR (OR = 0.94, 95% CI: 0.91–0.97, *p* < 0.001), PNI (OR = 0.88, 95% CI: 0.79–0.99, *p* = 0.030), and the Pre-CT AKI score (OR = 1.67, 95% CI: 1.29–2.16, *p* < 0.001) were significantly associated with CIN, while OPS (*p* = 0.149) and GPS (*p* = 0.879) were not significant.

In the multivariate analysis, baseline eGFR (OR = 0.96, 95% CI: 0.92–1.00, *p* = 0.081), PNI (OR = 0.82, 95% CI: 0.67–1.01, *p* = 0.067), and GPS (OR = 0.05, 95% CI: 0.00–0.65, *p* = 0.022) were included in the model. OPS (*p* = 0.224) and the Pre-CT AKI score (*p* = 0.154) were not statistically significant in the multivariate analysis. These results are presented in Table 7.

**Figure 2:**
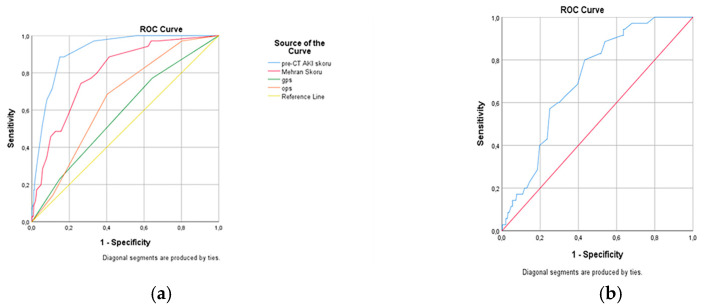
Receiver operating characteristic (ROC) curves illustrating the predictive performance of different models for contrast-induced nephropathy (CIN). (**a**) ROC curves of the Pre-CT AKI score, Mehran score, OPS, and GPS. The Pre-CT AKI score demonstrated the highest discriminative ability (AUC = 0.918), followed by the Mehran score (AUC = 0.800), while OPS and GPS showed limited predictive performance. (**b**) ROC curve of the Prognostic Nutritional Index (PNI), which was statistically significant but in the inverse direction (AUC = 0.764, *p* = 0.004).

The ROC analysis compared the predictive performance of different scoring systems for CIN. The Pre-CT AKI score demonstrated the highest discriminative ability (AUC = 0.918, 95% CI: 0.889–0.947, *p* < 0.001), with 89% sensitivity and 87% specificity. The Mehran score also showed good predictive performance (AUC = 0.800, *p* < 0.001), whereas OPS had limited predictive value (AUC = 0.654, *p* = 0.004). The PNI was statistically significant but exhibited an inverse association (AUC = 0.282, *p* = 0.020). In contrast, GPS did not reach statistical significance (AUC = 0.573, *p* = 0.207). Detailed results are presented in Table 8, and the corresponding ROC curves are illustrated in Figure 2a,b.

## 4. Discussion

In this study, the incidence of contrast-associated acute kidney injury (CA-AKI) among emergency department (ED) patients undergoing contrast-enhanced CT was 2.1%, consistent with the lower end of previously reported ranges (4–9%) depending on patient characteristics, comorbidities, and diagnostic definitions. Kim et al. (2011) observed an incidence of 4.5%; Traub et al. (2013) reported 6.7%; Huang et al. (2013) documented 8.6% in elderly patients; and Kooiman et al. (2010) noted 8.9% after CT pulmonary angiography [14,15,16,17]. More recently, Mosaddegh et al. (2025) reported an incidence of 8.8% in an ED-based cohort, consistent with this range [18]. McCullough (2008) previously highlighted the multifactorial pathophysiology of CA-AKI, involving contrast toxicity, oxidative stress, and renal hypoperfusion [19].

Meta-analyses support these findings, with Aycock et al. (2018) and Obed et al. (2022) showing that contrast-enhanced CT does not substantially elevate the risk of AKI in general populations [20,21]. In contrast, Hinson et al. (2017) emphasized that post-CT creatinine elevations are often multifactorial and not directly attributable to contrast exposure [22]. Similarly, Khodabandeh et al. (2024) and Long et al. (2025) highlighted that while the absolute risk of CA-AKI is low in most ED patients, high-risk subgroups such as those with advanced CKD, diabetes, or hemodynamic instability remain particularly vulnerable [23,24]. Kene et al. (2021) confirmed that ED patients with baseline CKD had a disproportionately higher risk, underscoring the importance of renal function in stratification [25]. Beyond the acute phase, CA-AKI has also been associated with higher rates of chronic kidney disease progression and increased long-term mortality, underscoring its broader clinical relevance [26].

Large-scale cohort studies have further clarified this risk stratification. B. Choi et al. (2025) demonstrated that contrast exposure did not substantially increase AKI incidence in the general ED population; however, the risk rose significantly when the baseline eGFR was below 45 mL/min/1.73 m^2^ [27]. McDonald et al. (2017), analyzing more than 150,000 ICU patients, reported that the risks of dialysis and mortality associated with contrast were limited to patients with impaired renal function [28]. Collectively, these findings underscore that CA-AKI should be viewed as a patient-specific rather than universal complication of contrast exposure. Within this context, our analysis revealed that among the evaluated scoring systems, the Pre-CT AKI score demonstrated the highest discriminative performance (AUC = 0.87), with excellent sensitivity and specificity. This aligns with the original validation study by Pattharanitima et al. (2025), which confirmed consistent accuracy across both emergency and inpatient cohorts [1]. Unlike conventional risk scores, the Pre-CT AKI model integrates easily obtainable clinical and laboratory parameters such as systolic blood pressure, serum creatinine, and hemoglobin, making it highly applicable in real-time ED workflows.

Collectively, these findings reinforce that CA-AKI risk in the ED is not uniform but conditioned by baseline kidney function and the burden of acute illness. The superior discrimination of the Pre-CT AKI score in our cohort supports a multifactorial, point-of-care approach rather than reliance on single-parameter screening. This aligns with recent perspectives that distinguish CA-AKI (associative) from contrast-induced injury (causal) and advocate risk-based, context-aware prevention in acute care pathways [2].

Beyond the established recommendation of optimal hydration, the current evidence highlights the importance of individualized, risk-adapted prevention strategies in contrast-associated acute kidney injury (CA-AKI). Integration of the Pre-CT AKI score into emergency workflows may enable early identification of susceptible patients and support personalized contrast dose adjustment, selection of iso- or low-osmolar contrast agents, and temporary discontinuation of nephrotoxic medications, in alignment with current ACR–NKF and KDIGO recommendations [29,30]. Additionally, immunonutritional evaluation using the Prognostic Nutritional Index (PNI) may aid in recognizing metabolically vulnerable individuals who could benefit from nutritional optimization and albumin supplementation, particularly in the context of systemic inflammation. The integration of risk-based prophylaxis, individualized post-contrast monitoring within 48–72 h, and emerging nephroprotective interventions—such as the endothelial and mitochondrial stabilization provided by SGLT2 inhibitors, Viggiano et al. (2025) may collectively enhance renal resilience and mitigate the burden of CA-AKI in high-risk populations [31]. Collectively, these multifactorial, precision-oriented measures expand the translational relevance of CA-AKI prevention beyond hydration alone.

Although the temporary discontinuation of renin–angiotensin system (RAS) inhibitors before contrast exposure is a common clinical practice, meta-analytic evidence indicates that such withdrawal does not consistently reduce the risk of contrast-associated acute kidney injury (CA-AKI). The pooled data show no significant difference in CIN incidence between continued and discontinued RAS blockade, suggesting that management should be individualized according to renal function and hemodynamic stability [29,32].

Interestingly, although the Pre-CT AKI score showed excellent discriminative performance in ROC analysis, it did not remain statistically significant in multivariate logistic regression. This discrepancy may be attributed to the relatively small number of CA-AKI events (n = 10) in our study, which limited the regression analysis’s statistical power and resulted in wide confidence intervals. Additionally, several pre-CT AKI components (creatinine, systolic blood pressure, and hemoglobin) were included as independent covariates, potentially introducing collinearity that attenuated their independent contribution. Similar results have been described previously, where multivariable adjustment reduced the predictive strength of composite indices in small ED cohorts [23,28]. This methodological difference between discrimination (ROC) and inference (regression) is acknowledged in sparse-event contexts; future research could use penalized likelihood methods or reduced-dimension models to address small-sample bias.

In contrast, the Mehran score, originally developed for patients undergoing percutaneous coronary intervention (PCI), demonstrated only moderate predictive ability in this ED-based cohort [5]. Although validated in interventional cardiology, its performance outside cardiac contexts appears limited. Abellás-Sequeiros et al. (2016) noted that the score tends to overestimate risk in non-PCI settings [33], while Sorgun et al. (2024) similarly found moderate utility but overestimation in high-risk subgroups [34]. These findings align with prior studies showing that PCI-derived scores may perform poorly in general or emergency populations [35].

The Prognostic Nutritional Index (PNI) is a simple immunonutritional biomarker calculated from serum albumin and lymphocyte count [13]. Consistent with Chang et al. (2023), who reported a 3.3-fold increased CIN risk with low PNI, and Yüksel & Köse (2022), who demonstrated an AUC of 0.87 in PCI cohorts [9,36], our findings suggest that nutritional and inflammatory status contribute significantly to renal vulnerability. This supports the broader use of immunonutritional indices in risk stratification beyond traditional renal markers.

Extending this concept, inflammation-based indices such as the systemic immune-inflammation index (SII) and systemic inflammation response index (SIRI) have been independently linked to CA-AKI [10], suggesting that simple hematologic ratios may capture systemic risk overlooked by renal indices alone. These results advocate for models integrating immunonutritional and clinical parameters—particularly relevant in the heterogeneous ED population.

By contrast, the Osaka Prognostic Score (OPS) and Glasgow Prognostic Score (GPS), which rely heavily on CRP and albumin levels, did not demonstrate significant predictive power in our cohort. While these indices have been validated in oncologic and cardiovascular cohorts [11,12,37], their limited utility in acute care likely reflects the variability of CRP and albumin in ED patients, where acute inflammatory conditions such as infection, trauma, and systemic inflammation are common confounders. Cheng et al. (2020) similarly reported that OPS and GPS performed inconsistently in acute cardiovascular cohorts, suggesting their limited transferability across clinical settings [38]. This highlights the importance of validating prognostic tools in the specific clinical populations where they are intended to be applied, rather than extrapolating from cohorts of chronic diseases.

In addition to conventional models, alternative scoring systems and machine learning (ML) approaches are being actively explored. Newer risk scores proposed by Duan et al. (2017), Lin et al. (2017), Kulkarni et al. (2023), and Yuan et al. (2022) have shown promising discrimination in high-risk cardiovascular patients, but their complexity, reliance on non-routine biomarkers, and lack of validation in ED settings limit their clinical applicability [39,40,41,42]. ML algorithms can incorporate high-dimensional data to capture nonlinear patterns. Lee et al. (2025) analyzed >22,000 ED cases and found that LightGBM achieved an AUROC of 0.731, with top predictors including creatinine, systolic blood pressure, albumin, eGFR, and hemoglobin [43]. Choi et al. (2024) similarly identified overlapping predictors—paralleling PNI and Pre-CT AKI [44].

However, despite their promise, ML approaches face practical barriers—particularly limited interpretability and challenges integrating with electronic health records. As emphasized by Khodabandeh et al. (2024), explainable AI (XAI) frameworks are essential to ensure clinician trust and real-world applicability [23].

Beyond scoring systems, neutrophil gelatinase-associated lipocalin (NGAL) may serve as an early marker for subclinical kidney injury, complementing risk models during rapid clinical decision-making [7]. A pragmatic ED approach could combine stage-aware stratification with selective biomarker testing in intermediate-risk patients, optimizing both resource allocation and diagnostic precision.

Recent guidelines and randomized trials support a multifactorial, individualized approach over universal prophylaxis. The **2024 KDIGO guidelines** discourage reliance on single thresholds such as eGFR and advocate for comprehensive evaluation including comorbidities, hemodynamic stability, and immunonutritional status [3]. The AMACING trial found that routine prophylactic hydration provided no additional benefit, even in high-risk groups, and was not cost-effective [45]. Likewise, consensus from the ACR–NKF recommends limiting intravenous hydration to patients with eGFR < 30 mL/min/1.73 m^2^ or established AKI [29]. Imaging optimization studies further support individualized contrast strategies. Varga et al. (2025) demonstrated that personalized CT pulmonary angiography protocols can minimize contrast dose without compromising image quality [43], and Barrios-López et al. (2021) confirmed the safety of low-dose regimens in patients with renal impairment [46].

Taken together, these findings underscore critical implications for ED practice. Although the overall risk of CA-AKI is relatively low, identifying high-risk individuals remains essential. The Pre-CT AKI score provides a robust, practical tool that outperforms traditional models such as Mehran. The PNI adds complementary prognostic value by reflecting the influence of immunonutritional status, while OPS and GPS appear less useful in acute contexts. Future risk stratification in ED settings should favor personalized, multifactorial strategies, potentially augmented by explainable ML models, to optimize both patient safety and resource efficiency.

Several limitations of this study should be acknowledged. First, its retrospective and single-center design may limit the generalizability of the findings. Although the overall sample size was relatively large, the number of patients who developed CA-AKI (n = 10; 2.1%) was small, which may have reduced the statistical power for subgroup analyses and contributed to the wide confidence intervals in some results.

Second, as in most CA-AKI studies, there is an inherent challenge in distinguishing kidney injury attributable to contrast exposure from injury caused by other factors such as sepsis, dehydration, or underlying comorbidities. Despite applying standardized criteria to exclude alternative explanations, residual confounding cannot be completely ruled out.

Third, while we evaluated several widely used risk scores—including the Mehran score, Pre-CT AKI score, PNI, OPS, and GPS—other emerging models and machine learning–based algorithms were not assessed. Future prospective, multicenter studies integrating traditional and machine learning–driven models could provide more comprehensive and individualized risk stratification.

Finally, this study did not investigate long-term outcomes such as the need for renal replacement therapy, progression to chronic kidney disease, or post-discharge mortality. Inclusion of these endpoints would offer greater insight into the prognostic implications of CA-AKI in emergency department populations.

## 5. Conclusions

In this study, the incidence of contrast-associated acute kidney injury (CA-AKI) was 2.1%, aligning with the lower end of rates reported in previous ED cohorts. Among the evaluated models, the Pre-CT AKI score demonstrated the best discriminative ability and emerged as the most reliable predictor of CA-AKI. The Mehran score showed moderate predictive performance, while OPS and GPS had limited clinical utility. The Prognostic Nutritional Index (PNI) was also independently associated with CA-AKI risk, suggesting that nutritional and inflammatory status contribute to renal vulnerability.

These findings highlight that CA-AKI risk assessment in emergency settings should not rely on single clinical or biochemical parameters, but rather on multivariable frameworks that integrate renal, hemodynamic, and immunonutritional markers. Future multicenter, prospective studies are warranted to validate these results and explore whether combining explainable machine learning models with established clinical scores can further enhance predictive precision and clinical applicability.

## Figures and Tables

**Table 1:** Summary of Risk Scoring Systems Evaluated in the Study.

Score	Included Parameters	Scoring Method	Points	Interpretation
**Mehran Risk Score** [5]	Hypotension,	Cumulative integer score derived from procedural and clinical variables	Hypotension = 5;	Low: <6; Moderate: 6–10; High: 11–15; Very high: ≥16
	IABP,		IABP = 5;	
	CHF (class III/IV),		CHF = 5;	
	Age > 75,		Age > 75 = 4;	
	Anemia,		Anemia = 3;	
	Diabetes,		Diabetes = 3;	
	Contrast Volume,		Contrast volume: +1 per 100 mL;	
	eGFR/Serum Creatinine		eGFR 40–60 = 2, <40 = 4, or Serum Cr > 1.5 mg/dL = 4	
**Pre-CT AKI Score** [1]	SBP,	Weighted logistic regression model for contrast-associated AKI	SBP < 90 mmHg = 2;	≥7 = high risk for CA-AKI
	Serum Creatinine,		Cr > 1.2 mg/dL = 2;	
	Hemoglobin,		Hb < 12 g/dL = 2;	
	eGFR,		eGFR < 60 = 3;	
	Contrast Volume		Contrast > 100 mL = 2 (0–12 total)	
**PNI (Prognostic Nutritional Index)** [13]	Albumin (g/dL), Lymphocyte count (/mm^3^)	Continuous index	PNI = (10 × albumin) + (0.005 × lymphocyte count)	Lower PNI = poor nutritional and immune status
**OPS (Osaka Prognostic Score)** [8]	CRP,	Inflammation- and nutrition-based composite score	CRP > 10 mg/L = 1;	Higher OPS = greater systemic inflammation
	Albumin,		Albumin < 3.5 g/dL = 1;	
	Total Lymphocyte Count		Lymphocytes < 1600/µL = 1 (0–3 total)	
**GPS (Glasgow Prognostic Score)** [12]	CRP, Albumin	Two-variable categorical score	CRP ≤ 10 mg/L and Albumin ≥ 3.5 g/dL = 0; either abnormal = 1; both abnormal = 2	Higher GPS = greater systemic inflammatory burden

**Table 2:** The baseline demographic and clinical characteristics of the study population.

Variable	Total (n = 479)	CIN (+) (n = 10)	CIN (−) (n = 469)	*p*-Value
Age, years	57.96 ± 19.16	79.70 ± 7.36	57.50 ± 19.06	<0.001
Male, n (%)	240 (50.1)	8 (80.0)	232 (49.5)	0.112
Female, n (%)	239 (49.9)	2 (20.0)	237 (50.5)	–
Heart rate, bpm	89.60 ± 19.40	98.80 ± 19.58	89.40 ± 19.37	0.119
SpO_2_ (%)	96.00 ± 3.91	94.60 ± 3.34	96.03 ± 3.92	0.062
Diabetes mellitus, n (%)	118 (24.6)	4 (40.0)	114 (24.3)	0.442
Hypertension, n (%)	168 (35.1)	5 (50.0)	163 (34.8)	0.506
Stroke, n (%)	21 (4.4)	2 (20.0)	19 (4.1)	0.098
Malignancy, n (%)	33 (6.9)	1 (10.0)	32 (6.8)	1.000

**Table 3:** Distribution of hospital outcomes according to CIN status.

Admission Status	CIN (−) (n = 469)	CIN (+) (n = 10)	Total (n = 479)	*p*-Value
Ward	322 (97.0%)	9 (3.0%)	332	0.184
Discharge	147 (99.3%)	1 (0.7%)	148	
**Total**	469	10	479	

**Table 4:** Distribution of CIN incidence according to CT type.

CT Type	CIN (−), n (%)	CIN (+), n (%)	Total	*p*-Value
Abdomen CT	310 (98.4%)	5 (1.6%)	315	**<0.001**
Pulmonary CT angiography	71 (93.4%)	5 (6.6%)	76	
Thoracic Aorta CT angiography	33 (100.0%)	0 (0.0%)	33	
Brain CT angiography	31 (100.0%)	0 (0.0%)	31	
Extremity Angio CT	19 (100.0%)	0 (0.0%)	19	
Brain CT	1 (100.0%)	0 (0.0%)	1	
Thorax CT	1 (100.0%)	0 (0.0%)	1	
**Total**	466 (97.9%)	10 (2.1%)	476	

**Table 5:** Distribution of CIN incidence according to selected primary diagnoses.

Diagnosis	CIN (−) (n)	CIN (+) (n)	Total (n)	CIN Incidence (%)
Heart failure	14	2	16	12.5%
Pneumonia	38	2	40	5.0%
Myocardial infarction	56	1	57	1.8%
Pulmonary embolism	47	1	48	2.1%
Cholecystitis	19	1	20	5.0%
Choledocholithiasis	12	1	13	7.7%
Ileus	23	1	24	4.2%
Urinary tract infection	17	1	18	5.6%
Other diagnoses	253	0	253	0.0%
**Total**	**479**	**10**	**489**	**2.1%**

**Table 6:** Laboratory findings in the total cohort and comparison between the CIN (+) and CIN (−) groups.

Variable	Total (n = 479) Mean ± SD	CIN (−) (n = 469) Mean ± SD	CIN (+) (n = 10) Mean ± SD	*p*-Value
Albumin (g/L)	38.38 ± 4.78	38.45 ± 4.79	35.10 ± 3.18	**0.028**
CRP (mg/L)	49.93 ± 63.09	49.68 ± 62.93	61.57 ± 73.04	0.556
Hemoglobin (g/dL)	12.67 ± 2.01	12.69 ± 2.01	11.98 ± 2.36	0.272
WBC (×10^9^/L)	11.12 ± 5.00	11.16 ± 5.02	9.00 ± 3.94	0.177
Neutrophils (×10^9^/L)	8.57 ± 4.83	8.60 ± 4.86	7.36 ± 3.76	0.425
Lymphocytes (×10^9^/L)	1.64 ± 0.92	1.65 ± 0.92	0.94 ± 0.68	**0.015**
PNI	38.39 ± 4.78	38.46 ± 4.79	35.10 ± 3.18	**0.028**
Baseline creatinine (mg/dL)	0.92 ± 0.28	0.92 ± 0.28	1.12 ± 0.24	**0.026**
Baseline eGFR (mL/min/1.73 m^2^)	83.85 ± 25.30	84.52 ± 25.04	52.20 ± 16.30	**<0.001**
Follow-up creatinine (mg/dL)	0.85 ± 0.32	0.83 ± 0.24	2.02 ± 1.07	**<0.001**
Follow-up eGFR (mL/min/1.73 m^2^)	89.71 ± 24.81	90.98 ± 23.39	29.90 ± 13.11	**<0.001**

**Table 7:** Univariate and multivariate logistic regression results for predictors of CIN.

Variable	Univariate OR (95% CI)	*p*-Value	Multivariate OR (95% CI)	*p*-Value
Baseline eGFR	**0.94 (0.91–0.97)**	**<0.001**	0.96 (0.92–1.00)	0.081
PNI	**0.88 (0.79–0.99)**	**0.030**	0.82 (0.67–1.01)	0.067
GPS	1.07 (0.42–2.73)	0.879	**0.05 (0.00–0.65)**	**0.022**
OPS	1.67 (0.83–3.35)	0.149	5.19 (0.61–44.18)	0.132
Pre-CT AKI score	**1.67 (1.29–2.16)**	**<0.001**	1.32 (0.92–1.89)	0.134

**Table 8:** ROC analysis of scoring systems for predicting CIN.

Score	AUC (95% CI)	Cut-off Value	Youden’s J	Sensitivity (%)	Specificity (%)	*p*-Value
Pre-CT AKI score	0.869 (0.795–0.943)	≥7	0.74	89	85	<0.001
Mehran score	0.838 (0.728–0.949)	≥6	0.48	74	74	<0.001
PNI	0.764 (0.674–0.854)	≤35	0.40	60	80	0.004
OPS	0.637 (0.499–0.776)	≥1	0.29	69	60	0.137
GPS	0.518 (0.349–0.687)	≥1	0.08	23	85	0.845

## Data Availability

The data presented in this study are available from the corresponding author upon request. The data is not publicly available due to ethical restrictions.

## Abbreviations

The following abbreviations are used in this manuscript:

AKI	Acute Kidney Injury
CA-AKI	Contrast-Associated Acute Kidney Injury
CIN	Contrast-Induced Nephropathy
CT	Computed Tomography
ED	Emergency Department
eGFR	Estimated Glomerular Filtration Rate
CKD	Chronic Kidney Disease
PNI	Prognostic Nutritional Index
OPS	Osaka Prognostic Score
GPS	Glasgow Prognostic Score
PCI	Percutaneous Coronary Intervention
ML	Machine Learning
XAI	Explainable Artificial Intelligence
KDIGO	Kidney Disease: Improving Global Outcomes

## References

[1] Pattharanitima P., Bumrungsong N., Phoompho B., Tanin R., Anumas S. (2025). Risk Score for Predicting Acute Kidney Injury from Contrast-Enhanced Computed Tomography (Pre-Computed Tomography Acute Kidney Injury Score) Training and Validation from Retrospective Cohort. Kidney360.

[2] Cho E., Ko G.J. (2022). The Pathophysiology and the Management of Radiocontrast-Induced Nephropathy. Diagnostics.

[3] Levin A., Ahmed S.B., Carrero J.J., Foster B., Francis A., Hall R.K., Herrington W.G., Hill G., Inker L.A., Kazancıoğlu R. (2024). Executive Summary of the KDIGO 2024 Clinical Practice Guideline for the Evaluation and Management of Chronic Kidney Disease: Known Knowns and Known Unknowns. Kidney Int.

[4] Wu M.J., Tsai S.F. (2022). Patients with Different Stages of Chronic Kidney Disease Undergoing Intravenous Contrast-Enhanced Computed Tomography—The Incidence of Contrast-Associated Acute Kidney Injury. Diagnostics.

[5] Mehran R., Aymong E.D., Nikolsky E., Lasic Z., Iakovou I., Fahy M., Mintz G.S., Lansky A.J., Moses J.W., Stone G.W. (2004). A Simple Risk Score for Prediction of Contrast-Induced Nephropathy after Percutaneous Coronary Intervention: Development and Initial Validation. J. Am. Coll. Cardiol..

[6] Bartholomew B.A., Harjai K.J., Dukkipati S., Boura J.A., Yerkey M.W., Glazier S., Grines C.L., O’Neill W.W. (2004). Impact of Nephropathy after Percutaneous Coronary Intervention and a Method for Risk Stratification. Am. J. Cardiol..

[7] Petrova I., Alexandrov A., Vladimirov G., Mateev H., Bogov I., Paskaleva I., Gotcheva N. (2023). NGAL as Biomarker of Clinical and Subclinical Damage of Kidney Function after Coronary Angiography. Diagnostics.

[8] Özbeyaz N.B., Algül E. (2024). A New Parameter in Predicting Contrast-Induced Nephropathy: Osaka Prognostic Score. Rev. Assoc. Med. Bras..

[9] Yuksel Y., Kose S. (2023). Prognostic Nutritional Index Predicts Contrast-Induced Nephropathy in Patients with Acute Coronary Syndrome. Angiology.

[10] Yildiz S.S., Cetinkal G., Kalendar E., Daglioglu E., Balaban B., Avsar M., Sit O., Aktas M., Kilickesmez K. (2025). Inflammatory Biomarkers Predicting Contrast-Induced Acute Kidney Injury in Elderly Patients with ST-Segment Elevation Myocardial Infarction. Diagnostics.

[11] Shibutani M., Maeda K., Nagahara H., Ohtani H., Iseki Y., Ikeya T., Sugano K., Hirakawa K. (2015). The Prognostic Significance of the Postoperative Prognostic Nutritional Index in Patients with Colorectal Cancer. BMC Cancer.

[12] Proctor M.J., Morrison D.S., Talwar D., Balmer S.M., O’Reilly D.S.J., Foulis A.K., Horgan P.G., McMillan D.C. (2011). An Inflammation-Based Prognostic Score (MGPS) Predicts Cancer Survival Independent of Tumour Site: A Glasgow Inflammation Outcome Study. Br. J. Cancer.

[13] Onodera T., Goseki N., Kosaki G. (1984). Prognostic Nutritional Index in Gastrointestinal Surgery of Malnourished Cancer Patients. Nihon Geka Gakkai Zasshi.

[14] Huang M.K., Hsu T.F., Chiu Y.H., Chiang S.C., Kao W.F., Yen D.H.T., Huang M.S. (2013). Risk Factors for Acute Kidney Injury in the Elderly Undergoing Contrast-Enhanced Computed Tomography in the Emergency Department. J. Chin. Med. Assoc..

[15] Kim K.S., Kim K., Hwang S.S., Jo Y.H., Lee C.C., Kim T.Y., Rhee J.E., Suh G.J., Singer A.J., Kim H.D. (2011). Risk Stratification Nomogram for Nephropathy after Abdominal Contrast-Enhanced Computed Tomography. Am. J. Emerg. Med..

[16] Kooiman J., Klok F.A., Mos I.C.M., Van Der Molen A., De Roos A., Sijpkens Y.W.J., Huisman M.V. (2010). Incidence and Predictors of Contrast-Induced Nephropathy Following CT-Angiography for Clinically Suspected Acute Pulmonary Embolism. J. Thromb. Haemost..

[17] Traub S.J., Kellum J.A., Tang A., Cataldo L., Kancharla A., Shapiro N.I. (2013). Risk Factors for Radiocontrast Nephropathy after Emergency Department Contrast-Enhanced Computerized Tomography. Acad. Emerg. Med..

[18] Mosaddegh R., Mohammadi F., Valipour A.M., Hosseini S.M., Naghshbandi M., Yarahmadi M., Faradonbeh N.A. (2025). The Incidence of Contrast-Induced Nephropathy Following Computed Tomography and Associated Risk Factors. Radiol. Res. Pract..

[19] McCullough P.A. (2008). Contrast-Induced Acute Kidney Injury. J. Am. Coll. Cardiol..

[20] Aycock R.D., Westafer L.M., Boxen J.L., Majlesi N., Schoenfeld E.M., Bannuru R.R. (2018). Acute Kidney Injury After Computed Tomography: A Meta-Analysis. Ann. Emerg. Med..

[21] Obed M., Gabriel M.M., Dumann E., Vollmer Barbosa C., Weißenborn K., Schmidt B.M.W. (2022). Risk of Acute Kidney Injury after Contrast-Enhanced Computerized Tomography: A Systematic Review and Meta-Analysis of 21 Propensity Score–Matched Cohort Studies. Eur. Radiol..

[22] Hinson J.S., Ehmann M.R., Fine D.M., Fishman E.K., Toerper M.F., Rothman R.E., Klein E.Y. (2017). Risk of Acute Kidney Injury After Intravenous Contrast Media Administration. Ann. Emerg. Med..

[23] Khodabandeh H., Ghahremani A.I., Gol A.Z., Jafari N., Soleimantabar H., Rabori V.S., Fooladi H., Noghabi F.A., Erfani A., Faramarzzadeh R. (2024). Contrast-Associated Acute Kidney Injury Following Intravenous Contrast Media Computed Tomography; New Concept and Future Directions: A Systematic Review Study on Emergencies Patients. J. Ren. Inj. Prev..

[24] Long B., Keim S.M., Gottlieb M., Schauer S.G., Schmitz G. (2025). Is Intravenous Contrast Associated with Increased Risk of Acute Kidney Injury?. J. Emerg. Med..

[25] Kene M., Arasu V.A., Mahapatra A.K., Huang J., Reed M.E. (2021). Acute Kidney Injury after Ct in Emergency Patients with Chronic Kidney Disease: A Propensity Score-Matched Analysis. West. J. Emerg. Med..

[26] Weisbord S.D., Palevsky P.M. (2011). Contrast-Induced Acute Kidney Injury: Short- and Long-Term Implications. Semin. Nephrol..

[27] Choi B., Heo S., Mcdonald J.S., Choi S.H., Choi W.M., Lee J.B., Lee E.A., Park S.H., Seol S., Gan S. (2025). Risk of Contrast-Induced Acute Kidney Injury in Computed Tomography: A 16 Institutional Retrospective Cohort Study. Investig. Radiol..

[28] McDonald J.S., McDonald R.J., Williamson E.E., Kallmes D.F., Kashani K. (2017). Post-Contrast Acute Kidney Injury in Intensive Care Unit Patients: A Propensity Score-Adjusted Study. Intensive Care Med..

[29] Davenport M.S., Perazella M.A., Yee J., Dillman J.R., Fine D., McDonald R.J., Rodby R.A., Wang C.L., Weinreb J.C. (2020). Use of Intravenous Iodinated Contrast Media in Patients with Kidney Disease: Consensus Statements from the American College of Radiology and the National Kidney Foundation. Radiology.

[30] Chen J.J., Lee T.H., Chan M.J., Tsai T.Y., Fan P.C., Lee C.C., Wu V.C., Tu Y.K., Chang C.H. (2024). Electronic Alert Systems for Patients with Acute Kidney Injury: A Systematic Review and Meta-Analysis. JAMA Netw. Open.

[31] Viggiano D., Joshi R., Borriello G., Cacciola G., Gonnella A., Gigliotti A., Nigro M., Gigliotti G. (2025). SGLT2 Inhibitors: The First Endothelial-Protector for Diabetic Nephropathy. J. Clin. Med..

[32] Wang W., Qu W., Sun D., Liu X. (2020). Meta-Analysis of Effect of Renin–Angiotensin–Aldosterone System Blockers on Contrast-Induced Nephropathy. J. Renin-Angiotensin-Aldosterone Syst..

[33] Abellás-Sequeiros R.A., Raposeiras-Roubín S., Abu-Assi E., González-Salvado V., Iglesias-Álvarez D., Redondo-Diéguez A., González-Ferreiro R., Ocaranza-Sánchez R., Peña-Gil C., García-Acuña J.M. (2016). Mehran Contrast Nephropathy Risk Score: Is It Still Useful 10 Years Later?. J. Cardiol..

[34] Sorgun O., Karaali R., Arıkan C., Kanter E., Yurtsever G. (2024). Emergency CT Scans: Unveiling the Risks of Contrast-Associated Acute Kidney Injury. Tomography.

[35] Ma B., Allen D.W., Graham M.M., Har B.J., Tyrrell B., Tan Z., Spertus J.A., Brown J.R., Matheny M.E., Hemmelgarn B.R. (2019). Comparative Performance of Prediction Models for Contrast-Associated Acute Kidney Injury after Percutaneous Coronary Intervention. Circ. Cardiovasc. Qual. Outcomes.

[36] Chang W.T., Sun C.K., Wu J.Y., Huang P.Y., Liu T.H., Chang Y.J., Lin Y.T., Kang F.C., Hung K.C. (2023). Association of Prognostic Nutritional Index with Risk of Contrast Induced Nephropathy: A Meta-Analysis. Front. Nutr..

[37] Feng J., Wang L., Wang L., Yang X., Lou G. (2022). Clinical Significance of Osaka Prognostic Score Based on Nutritional and Inflammatory Status in Patients with Esophageal Squamous Cell Carcinoma. BMC Cancer.

[38] Cheng E.L., Hong Q., Yong E., Chandrasekar S., Tan G.W.L., Lo Z.J. (2020). Validating the Use of Contrast-Induced Nephropathy Prediction Models in Endovascular Aneurysm Repairs. J. Vasc. Surg..

[39] Duan C., Cao Y., Liu Y., Zhou L., Ping K., Tan M.T., Tan N., Chen J., Chen P. (2017). A New Preprocedure Risk Score for Predicting Contrast-Induced Acute Kidney Injury. Can. J. Cardiol..

[40] Lin K., Zheng W., Bei W., Chen S., Islam S.M., Liu Y., Xue L., Tan N., Chen J. (2017). A Novel Risk Score Model for Prediction of Contrast-Induced Nephropathy after Emergent Percutaneous Coronary Intervention. Int. J. Cardiol..

[41] Kulkarni C., Kothari J., Sirsat R., Almeida A. (2023). A Simplified Risk Score to Estimate the Risk of Contrast-Induced Nephropathy after Contrast Exposure. Indian J. Nephrol..

[42] Yuan Y., Qiu H., Xiaoying H., Zhang J., Wu Y. (2022). A Risk Score Model of Contrast-Induced Acute Kidney Injury in Patients with Emergency Percutaneous Coronary Interventions. Front. Cardiovasc. Med..

[43] Lee K., Jung W., Jeon J., Chang H., Lee J.E., Huh W., Cha W.C., Jang H.R. (2025). Prediction of Contrast-Associated Acute Kidney Injury with Machine-Learning in Patients Undergoing Contrast-Enhanced Computed Tomography in Emergency Department. Sci. Rep..

[44] Choi H., Choi B., Han S., Lee M., Shin G.T., Kim H., Son M., Kim K.H., Kwon J.M., Park R.W. (2024). Applicable Machine Learning Model for Predicting Contrast-Induced Nephropathy Based on Pre-Catheterization Variables. Intern. Med..

[45] Nijssen E.C., Rennenberg R.J., Nelemans P.J., Essers B.A., Janssen M.M., Vermeeren M.A., van Ommen V., Wildberger J.E. (2017). Prophylactic Hydration to Protect Renal Function from Intravascular Iodinated Contrast Material in Patients at High Risk of Contrast-Induced Nephropathy (AMACING): A Prospective, Randomised, Phase 3, Controlled, Open-Label, Non-Inferiority Trial. Lancet.

[46] Barrios López A., García Martínez F., Rodríguez J.I., Montero-San-Martín B., Gómez Rioja R., Diez J., Martín-Hervás C. (2021). Incidence of Contrast-Induced Nephropathy after a Computed Tomography Scan. Radiologia (Engl. Ed.).

